# Bridging the Gap Between Artificial Intelligence Understanding and Clinical Implementation in Cardiovascular Medicine: A Commentary on Heinrich and Voigt's Review

**DOI:** 10.1002/clc.70185

**Published:** 2025-07-23

**Authors:** Shaher Yar, Keshav R. Baskaran, Aarushi Mishra, Pratiksha Paudel

**Affiliations:** ^1^ University College of Medicine and Dentistry Lahore Pakistan; ^2^ Mount Multispeciality Hospital Chennai India; ^3^ Danylo Halytaky Lviv National Medical University Lviv Ukraine; ^4^ Department of Internal Medicine All Nepal Hospital Kathmandu Nepal

To the Editor,

The comprehensive analysis by Heinrich and Voigt on artificial intelligence (AI) concepts in cardiovascular medicine has piqued our interest [1]. Their methodical approach to clarifying AI principles provides an essential foundation for physicians' knowledge and understanding of AI in clinical practice. However, we believe that the discussion would benefit from addressing significant implementation gaps beyond theoretical understanding. It is essential to consider the practical challenges that hinder the adoption of AI in routine cardiovascular practice.

While inadequate understanding and mistrust among physicians certainly pose barriers to the acceptance of AI, institutional infrastructure constraints represent equally significant challenges. Many healthcare systems still lack the robust technical infrastructure, effective data standardization procedures, and cohesive regulatory frameworks necessary for the seamless integration of AI [2]. This is particularly concerning in resource‐limited environments, where the potential benefits of AI could be most transformative. For healthcare managers and physicians contemplating AI integration into their clinical practice, the fragmented regulatory landscape, exacerbated by rapidly changing FDA approval procedures for AI‐enabled medical devices, adds another layer of uncertainty.

Validation problems in cardiovascular AI models require special attention. While many of these models demonstrate impressive performance in retrospective studies, they often struggle to produce generalizability in prospective studies in clinical settings. Although controlled studies indicate that 68.75% of AI interventions lead to clinical improvements compared to human experts, only 17.2% of these interventions have been subjected to randomized controlled trials [3]. This highlights a significant gap in evidence. The discrepancy between theoretical potential and actual performance underscores the need for robust external validation systems that take into account institutional variations, demographic diversity, and differences in equipment.

The authors rightly emphasize the importance of physician education and understanding of AI integration. However, successful implementation requires coordinated and multidisciplinary strategies. Currently, AI development is often driven by industry silos that prioritize commercial viability over clinical effectiveness. The lack of physician involvement in AI training and validation has resulted in models that, despite theoretical promise, lack significant clinical benefits [4]. Additionally, the complexity of these models, often described as “black boxes,” undermines physician confidence and complicates medical decision‐making, particularly in terms of accountability [4].

In addition to technical issues, it's important to consider the financial implications of integrating AI. Smaller institutions and underprivileged areas, in particular, face significant challenges due to high computational costs and infrastructure requirements. While these settings could greatly benefit from enhanced diagnostic and prognostic capabilities, they often lack the necessary tools for full integration of AI.

These findings led us to propose four key recommendations to enhance the use of AI in cardiovascular medicine. First, we urge the development of comprehensive implementation strategies that address technical, regulatory, and workflow integration across diverse healthcare settings. Second, establishing collaborative validation systems is essential for thorough external testing of AI models across various companies and patient populations. Third, we recommend targeted physician education initiatives that combine practical experience with theoretical knowledge to boost confidence in AI‐assisted decision‐making. Finally, we call for rigorous post‐market surveillance of AI systems in cardiovascular practice along with streamlined regulatory processes for quicker approvals.

The transition from AI research to clinical application requires a systematic approach to address various challenges. While Heinrich and Voigt's study provides a crucial theoretical foundation, successful integration of AI also hinges on overcoming practical implementation barriers. Recognizing these challenges and developing comprehensive solutions will enable the cardiovascular community to fully leverage the transformative power of AI and ensure equitable access across various healthcare settings.

## Conflicts of Interest

The authors declare no conflicts of interest.

## Data Availability

The authors have nothing to report.
